# Presuming the influence of the media: teenagers′ constructions of gender identity through sexual/romantic relationships and alcohol consumption

**DOI:** 10.1111/1467-9566.12107

**Published:** 2014-01-21

**Authors:** Jane E K Hartley, Daniel Wight, Kate Hunt

**Affiliations:** 1 Child and Adolescent Health Research Unit, School of Medicine, University of St Andrews; 2 Medical Research Council, Chief Scientist Office, Social and Public Health Sciences Unit, University of Glasgow

**Keywords:** teenagers, media influence, drinking alcohol, gender identity, sexual relationship

## Abstract

Using empirical data from group discussions and in-depth interviews with 13 to 15-year olds in Scotland, this study explores how teenagers’ alcohol drinking and sexual/romantic relationships were shaped by their quest for appropriate gendered identities. In this, they acknowledged the influence of the media, but primarily in relation to others, not to themselves, thereby supporting Milkie's ‘presumed media influence’ theory. Media portrayals of romantic/sexual relationships appeared to influence teenagers’ constructions of gender-appropriate sexual behaviour more than did media portrayals of drinking behaviour, perhaps because the teenagers had more firsthand experience of observing drinking than of observing sexual relationships. Presumed media influence may be less influential if one has experience of the behaviour portrayed. Drinking and sexual behaviour were highly interrelated: sexual negotiation and activities were reportedly often accompanied by drinking. For teenagers, being drunk or, importantly, pretending to be drunk, may be a useful way to try out what they perceived to be gender-appropriate identities. In sum, teenagers’ drinking and sexual/romantic relationships are primary ways in which they do gender and the media's influence on their perceptions of appropriate gendered behaviour is mediated through peer relationships.

## Introduction

In comparison to other western European nations, the UK has a poor record of sexual health in teenagers. Approximately half of all sexually transmitted infections diagnosed in the UK in 2009 were seen in the under-25s. In most high-income countries, including the UK, the age at which teenagers have their first sexual experience is falling, teenagers′ alcohol consumption is increasing (Currie *et al*. 2012), and the number of young people experiencing alcohol-associated morbidity and mortality is rising. Furthermore, there is increasing evidence that health-related practices such as drinking are linked to performance of gender in young adults (de Visser and Smith 2007). Understanding the factors underlying alcohol consumption and sexual activity among teenagers is therefore an important public health priority. This article explores the possible influence of the media on teenagers′ constructions of gendered identities, with a specific focus on drinking alcohol and engaging in sexual and/or romantic relationships. First, we provide an overview of the following themes: how media influence young people, including the formation of their gendered identities; how gendered identities are constructed; and how they are expressed through the use of alcohol and sexual and romantic relationships. We then describe our research methods. Third, the findings are presented in relation to the following aspects of gender identity: teenagers′ use of the media in general; their sexual and romantic relationships and the media portrayals of these; and their alcohol-drinking, and the media portrayals of this. Finally, we explore the relevance of these findings for Milkie's theory of presumed media influence. Throughout, it should be noted that we use the term gender-appropriate in relation to teenagers′ attempts to explore what kind of behaviour conforms to the perceived gender norms of their (localised) social groups. Our position is that the participants in our study are attempting to align themselves with a specific group by constantly negotiating what they understand to be appropriate gendered behaviour.

## Media influence

There are two opposing theoretical positions on the influence of the media: the media as powerful versus the audience as powerful. The evidence that media are powerful*,* and that they contribute to teenagers′ behaviour, is substantial. Half a century of research shows that the media can have an impact on virtually every public health concern about teenagers. These include early sexual activity (Strasburger *et al*. 2009), alcohol consumption (Hanewinkel *et al*. 2012), illegal drug use (Borzekowski and Strasburger 2008), aggressive behaviour (Strasburger *et al*. 2009), obesity (Gantz *et al*. 2007) and eating disorders (Strasburger *et al*. 2009). However, all these studies are quantitative, and most originate from the USA.

The literature supporting the theory that the media audience is powerful is largely qualitative and UK-based (see Buckingham and Bragg 2004, Buckingham 2007, Livingstone 2002 2009, Lyons *et al*. 2006). The active audience concept pertains directly to research on how audiences receive media messages. In particular, Hall (1973) suggests that media texts are open to interpretation by individual viewers who bring their own experience and critical faculties to them. Similarly, readers of media texts rarely take up the stories offered to them directly (Hodgetts and Chamberlain 2003). Rather, they engage and interact with media content, drawing on it selectively for particular purposes, accepting, rejecting, resisting and modifying representations to suit their own particular purposes. Gerbner *et al*. (2002) state that, while understandings are circulated widely through the media, caution is required in reaching conclusions about how the media exert effects on people.

In the early 1980s Davison (1983) argued that individuals tend to perceive that the mass media have a greater influence on others than on themselves. Following extensive research on this third-person effect, Gunther and Storey (2003) developed a comprehensive model of indirect media effects. This model, termed the influence of presumed influence, derives from the idea that individuals will perceive, and presume that the media have some influence on others and will change their own attitudes or behaviour accordingly. A seminal work in this area is Milkie's (1999). Although she does not refer to the influence of presumed influence, a term coined later by Gunther and Storey (2003), this is essentially the process that she identifies and so we will refer to Milkie's (1999) presumed media influence theory. Milkie's study was an attempt at understanding how American girls may be affected by the prominent female images pervasive in girls′ magazines. The girls in her study generally understood that these media images were unrealistic (Milkie 1999: 199) but clear ethnic differences were observed among their responses. White girls reported feeling negatively affected by them since they believed that their peers, and particularly boys, thought that the images were important and that they evaluated girls on the basis of these images. Their recognition that the images were unrealistic was therefore overridden by the anticipated social comparison of peers (Milkie 1999: 201). Ethnic minority girls, however, did not identify with white media images, nor did they believe that their significant others were influenced by them and so they did not feel negatively affected by the images. Essentially, the mainstream female images, although they were mostly criticised as unrealistic, became an oppressive negative referent for white girls who could not escape them easily, but not for black girls, who felt distant from them (Milkie 1999: 199).

Milkie concludes that individuals believe that others are more strongly affected by media portrayals than they are themselves. A complex, indirect effect may also occur as people account for the effects of the pervasive imagery in the media on others in their social networks, and are themselves influenced by perceptions of the way others see the media-distorted world (Milkie 1999: 193-4). In sum, the presumed media influence model reconciles, to some extent at least, the tension between the powerful media and the powerful audiences′ positions. We will now briefly explore the construction of gendered identity before turning to the two health-related kinds of behaviour at issue.

## The construction of gendered identities

Identities are how we understand who we are. Individuals become aware of their identities through their relationships with some external factor, often another person, which is conceptualised in the seminal theory of symbolic interactionism. Cooley's (1964) looking-glass self suggests that people must consider themselves through the eyes of others, and in doing so they can understand and attain their own social identity (McIntyre 2007). A central component of identity in most cultures is gender, and so it is especially difficult for many teenagers as they shift from homosocial to more heterosocial peer groups (Gagnon and Simon 1974) and increasingly negotiate their identities with a different audience, namely the opposite sex. The performative aspect of gender has received ever more attention in social research (Butler 2004, West and Zimmerman 1987). Rather than being viewed as two static (biological) categories – male and female – gender is a ‘set of socially constructed relationships which are produced and reproduced through people's actions’ (Gerson and Peiss 1985: 12). Gender is something one does, and does recurrently, in interaction with others.

Two important ways in which teenagers and young adults in western countries construct their gendered identities are through the practices of drinking alcohol and of engaging in sexual/romantic relationships. They demonstrate particular femininities and masculinities in particular socio-cultural settings and interactions (Campbell 2000, Courtenay 2000, Maclean *et al*. 2010, Marston and King 2006, Mullen *et al*. 2007, Peralta 2007, Wight 1994). Many sociocultural factors are associated with, and influence, health-related behaviour but gender, Courtenay (2000) argues, is one of the most important.

## Drinking alcohol as a performance of gendered identities

The masculine dimensions of drinking alcohol have long been documented (*see* Dennis *et al*. 1956) and more recently confirmed by De Visser and Smith (2007), who found that many young men believe that drinking, and being able to ‘hold’ one's drink, are important components of masculinity. They also showed that men, in constructing their masculinities, traded drinking competence with competence in other areas, such as excelling in sport (de Visser and McDonnell 2013, de Visser and Smith 2007: 598). However, although gender differences in consumption persist, in Scotland (Emslie *et al*. 2009) and in most countries (Wilsnack *et al*. 2000) women are increasingly engaging in more traditionally masculinised practices such as binge-drinking and aggressive behaviour. For women, drinking alcohol has become a means of pursuing contemporary femininities (Day *et al*. 2003, 2004, Pavis *et al*. 1997).

## Sexual and romantic relationships as a performance of gendered identities

Heterosexual sexual and romantic relationships are by definition gendered. As Weeks (1986) has noted, although they are intimately personal and often private, they also have important social dimensions, the constitution of gendered identity being one. Globally, stereotyped versions of gender-appropriate sexual behaviour still prevail: men are expected to be highly heterosexually active and women to be interested in sex only within defined relationships, which vary culturally (Marston and King 2006). By and large, in Britain vaginal penetration is an important symbol of masculinity and of the transition from boyhood to manhood (Wight 1994), while for young women sex without romance is ‘unfeminine’ (Holland *et al*. 1998). However, as with drinking, these gender norms have weakened to some extent in recent decades. For example, Forrest (2010) has shown how young men can reconstruct their masculine identities through their commitment to ‘serious’ relationships, while for young women it is considered less intrinsically unfeminine to initiate sexual encounters, and same-sex relationships are increasingly acceptable. That said, we draw on the accounts of Scottish teenagers to address the following questions: to what extent did the media feature in these teenagers’ lives; did they think media portrayals of sexual/romantic relationships and drinking influenced their own, or their peers’, behaviour; and how did this behaviour contribute to their construction of their gendered identities? In the discussion we return to Milkie's theory of presumed media influence and how it might be developed in the light of our findings.

## Methods

Qualitative methods were deemed the most appropriate way of exploring teenagers’ understandings of their own and their peers’ use of the media and of alcohol, and sexual/romantic relationships. The participants were 13 to 15-year olds from two urban schools in the east and west of Scotland: ‘Rosefield High’ in Edinburgh and ‘Yatesly Academy’ in the west of Scotland. These schools were selected from those participating in an evaluation of the Healthy Respect sexual health programme (Elliott *et al*. 2013). Both schools included pupils from a wide socioeconomic catchment area. See Table1.

**Table 1:** Composition of group discussions and follow-up interviews

Group identified	Participants′ pseudonyms	Individual/paired interviews
Mxd. Ro. 3a (S.3)	Jordan, Caroline and Daisy	None
Mxd. Ro. 3b (S.3)	Lorna, Stephanie, Robert and Josh	None
Mxd. Ro. 4a (S.4)	Frank, Heidi, Louise and Kristofer	Frank (II): follow-up individual interview Heidi (II): follow-up individual interview
Mxd. Ro. 4b (S.4)	Robert, Andrew, Jessie, Julia	None
B.Ro.4a (S.4)	Jonathan, Cameron, Dougie, Euan, Ferg, James	None
B.Ro.4b (S.4)	Ritche, Aaron, Robin	None
B.Ro.4c (S.4)	Dillon, Jake, Ewan	None
G.Ro.4a (S.4)	Milly, Molly, Cheryl, Gail	Milly and Molly (paired interview)
G.Ro.4b (S.4)	Catherine, Eva, Joanne, Sally	Eva (II): follow-up individual interview
G.Ro.4c (S.4)	Sasha, Kelly, Miquette	None
S4 Personal contact; not affiliated to either school		Rhona (II)
S4; Personal contact; not affiliated to either school		Lucy (II)
B.Ya.3a (S.3)	Tom, Bruce, Calum, Darren	None
B.Ya.3b (S.3)	Thomas, Mark, Carl, Gavin	Carl (II); individual interview
B.Ya.3c (S.3)	Mike, Norman, Eric, Dave	Dave (II); individual interview Norman (II) individual interview
B.Ya.4a (S.4)	Bryn, Harvie, Greg	None
B.Ya.3d (S.3)	Johnny, George, Iain	Johnny (II); individual interview
B.Ya.3e (S.3)	Adam, Ross, Martin, Kai	Kai (II); individual interview
B.Ya.3f (S.3)	Ally, Richie, Chris, Sandy	None
G.Ya.3a (S.3)	Alison, Kat, Lynsey	None
G.Ya.3b (S.3)	Sarah, Kate, Susan, Jill	None
G.Ya.3c (S.3)	Carly, Holly, Laura, Danielle	None
G.Ya.3d (S.3)	Anna, Karly, Louise, Hannah	None
G.Ya.3e (S.3)	Natalie, Val, Lynne	None

**Note:** Participant code abbreviations: sex; school; age group. For example, B.Ro.4b means boys, Rosefield, secondary year 4 The lower-case letters a to f refer to the number of groups in that category. Those who took part in individual interviews are identified by (II) after their name.

B, boys, G, girls, mxd., mixed sex; R, Rosefield School, Ya, Yately Academy.

Between 2007 and 2009, group discussions and subsequent individual interviews with the group discussion participants were undertaken. Group discussions were conducted on school premises; individual interviews were conducted either on school premises or in the participant's home. All were audio-taped, with the participants′ permission. Where possible, friendship groups were recruited to permit group discussion participants to be as open and relaxed as possible. Pseudonyms are used for all participants and schools, and all transcriptions were anonymised. The study was approved by the Ethics Board of the Faculty of Law, Business and Social Sciences at the University of Glasgow, and conformed to the relevant child protection guidelines. These required that if, during the interviews or group discussions, participants under the age of 16 disclosed that they may be at serious harm from others, we were required to pass on this information to the appropriate authority. This was made clear to participants at the start of the group discussion or interview. During only one of the group discussions did a participant disclose information of an incident which we felt did put him at risk, and this was passed on to the relevant teachers.

The group discussions and individual interviews included sensitive topics such as sexual behaviour, smoking, drinking and illegal drug-use. Of interest was how presentations of this behaviour in the media were interpreted by the participants and how this related to the way that participants talked about this behaviour. The disclosure of potentially sensitive information – for example, on the participants′ personal sexual history – was discouraged. Rather, the intent was to learn more about their use and interpretations of the media more generally, or their perceptions and expectations of others′ behaviour. The group discussions were particularly valuable for observing directly how gendered identities were performed between peers. Before each group discussion, the importance of respecting confidentiality in relation to issues discussed within the group was stressed. The individual interviews allowed the participants to talk about more personal and sensitive aspects of their lives, if they wished to, which were inappropriate for group discussions, especially romantic/sexual relationships. They were of particular benefit in exploring the complex and varied ways in which teenagers both understood and articulated the concept of media influence.

Towards the end of the group discussions and individual interviews, time permitting, the participants were asked to reflect on selected images from contemporary British TV programmes *Skins*,* Hollyoaks* and *Shameless*.^1^ Because *Skins* and *Shameless* were classed as suitable only for teenagers older than our participants, we selected still images (rather than video clips) to prompt discussion. These images were used to prompt discussion about what forms of alcohol use and sexual/romantic behaviour were considered gender-appropriate. Following Milkie's conceptual approach to explore whether or not teenagers integrated media portrayals into their own notions of gender-appropriate behaviour, the participants were questioned about their presumptions of what the opposite sex expected of them and of the origins of these expectations. For example, with reference to girls′ sexual and romantic relationships, the following matters were addressed: their reported experiences and expectations of romantic/sexual relationships; their perceptions of where these originated; their assumptions about what boys thought that girls would want from a relationship with a boy; and, finally, their assumptions about where boys presumed the girls′ expectations came from. Similarly, equivalent lines of enquiry were pursued in exploring boys′ sexual and romantic relationships. The media content being reflected upon and interpreted was largely what participants had said they had watched.

Transcripts were read and re-read to allow the identification of initial themes which were then coded using the software package NVivo 7. These themes were discussed among all authors on the basis of their own independent reading of the data. Broad codes were noted initially to categorise the content of the transcripts, and these codes were saved in NVivo7 as tree nodes: for example, presumed media influence, norms of sexual relationships or obvious gender presentation. This revealed the how nodes were interlinked or related to the transcripts. The meanings of the tree nodes were then summarised by accessing a particular node and manually summarising the emergent ideas. Connections were identified among themes and a number of overarching themes emerged: gender, general media, TV programmes (that is, specific data referring to *Skins*,* Hollyoaks* and *Shameless*), sexual/romantic relationships, emotions, identity/presentations of self, and alcohol. This process involved detecting patterns of associations within the data. Below we present findings from this analysis. The data are structured as follows: (i) the participants′ reported general use of media; (ii) gendered accounts of expectations of romantic and sexual relationships, of direct media influence on their romantic and sexual relationships, of presumed media influence on their romantic and sexual relationships; and (iii) media portrayals of drinking and sex.

## Reported general use of media

Media use was part of daily routine. Even those who claimed not to be interested in the media (often boys) would later reveal that they had indeed spent time playing computer games, watching sport on TV or communicating with friends via social media. Participants often displayed in-depth knowledge of the characters and storylines in *Hollyoaks*,* Skins* and *Shameless*. Media-based talk revealed how they belonged or did not belong to a group. A typical response to the question: ‘What do you watch?’ was Bryn′s: ‘What everyone else is watching’ (B.Ya.4a). Not to know the latest storyline in a popular series could exclude them from conversations. Talk of media could be used as a point of connection:

Calum: All I hear girls talking about nowadays, well since it started, is *90210*^2^ and how it's happening and what's happening and that. ‘Coz, like, it's on tonight and everyone′ll come back in tomorrow and go, ‘Oh did you see that?’
Bruce: That's what happened with *Big Brother* and that.
Calum: Aye exactly.
Darren: It makes conversation, things like that so … Something to talk about, instead of saying the same thing all the time.
Calum: It's like you′re afraid of them [girls]. Well you′re no afraid but it's like, you′re not confident enough, but then you start talking about that and you start talking about it every week. And they might get fed up of that after a while … Then you have to think of something to talk about, and you′re like, ‘oh no’. [Laughs] (B.Ya.3a)

This shared media-talk is at the crux of the presumed media influence model: that is, for media to be influential participants must presume – whether accurately or not – which media others, particularly the opposite sex, are using. For example, a boy can presume a girl watches certain content, presume it affects her, and the boy acts accordingly, irrespective of what she does indeed watch. Boys typically reported knowing which television soaps their female acquaintances watched; the girls typically talked of boys watching pornography.

## Gendered accounts: expectations of romantic and sexual relationships

What expectations did the teenagers have of romantic and sexual relationships? Take these typical girls’ expectations of relationships: Eva (II) wanted ‘a long-lasting, mature relationship. A couple should be thoughtful and they *should* argue’; Lucy (II) sought ‘respect. No cheating, no violence, no secrets’; while Rhona (II) thought a relationship should last a long time, and that the couple should hold hands and have long conversations. In many respects this was mirrored by boys’ ideas about what girls expected. They presumed girls wanted exclusivity: that is, the boys should not see anyone else, including even their male friends. On the other hand, girls assumed that boys wanted a relatively short relationship: ‘for a few weeks’ (Heidi-II); ‘they do not want a long term relationship, all they want to do is brag to their friends’ (Alison and Kat; G.Ya.3a) and they will ‘probably be bored after a short while’ (Kate and Susan; G.Ya.3b), a view not wholly supported by the boys. Some male participants (B.Ya.3a, B.Ya.3c and B.Ya.4a) expected that their next relationships would be exclusive since ‘going with someone else seems to annoy them [girls]’ (Tom B.Ya.3a).

Girls assumed that, for boys, sex was to the fore: [they wanted someone] ‘short, sweet, shaggable. All guys are the same’ (Lauren; G.Ya.3c). Alison (G.Ya.3a) concurred: ‘A boy wants sex’. Dylan appeared to conform to the stereotype:

Dylan: the girl fair.
I: Treat the girl fair?
Dylan: Aye.
I: Okay. What does that mean exactly?
Dylan: Get into their pants, basically. (Laughter)
I: Right.
Dylan: No, have respect for the bitch. (Laughter)

Most girl participants commented that boys would expect them to be sexually active. When asked about her future relationship, Rhona (II) said ‘I don′t think I′d take it very well with someone forcing you into doing something. Like, I′ve had that before and I wasn′t happy, and I ended it’. In this context, we understand this ‘something’ to mean some kind of sexual behaviour. Her uncomfortable past experiences were informing her expectations.

## Gendered accounts: direct media influence on romantic and sexual relationships

Few participants – slightly more girls than boys – reported that the media directly influenced their relationships. Of the boys, only Kai (II) said he personally was directly influenced by the media. In his case it was about relationship expectations when he said that he looked to Ross and Rachel in *Friends* as role-models. Frank (II) said that it is possible that boys are *‘*egged on by media about looks and being popular’. He was comparing this attitude with girls, who were more likely to be influenced ‘about emotions, being in a relationship and being in love’. Participants in B.Ro.4b confirmed the girl participants’ statements that ‘lots of boys watch porn, men too’. They envisaged that boys could learn about sex from watching pornography. The boys’ views on girls’ greater susceptibility to direct media influence were given partial support by the girls. Two specific programmes were said to be directly influential: the US romantic drama *Beverly Hills 90210* and *Skins*. Girls reported that they would be more influenced if media portrayals were more like real life. They also said that expectations which they took from the media often led to disappointment in real life. For example, Sarah (G.Ya.3b) lamented:

Like, they [TV portrayals of relationships with boys] always say the boy will come running after you and that won′t actually happen and you′ve got, somehow you′ve got this into your head that it's gonna happen, but like, you have to just accept that it's not. It's really hard to do that.

In her interview, Eva used the media to make sense of her relationship:

I: Where do you think you got these expectations (about relationships) from?
Eva: From romantic movies probably [laughs].
I: Really? So what is it, tell me a bit more about that?
Eva: Right, in a movie like *The Notebook* and stuff like, they just do like anything to be with each other and stuff. So something like that.
I: And is that something that you′d want to do, is that your kind of ideal relationship?
Eva: Yeah.
I: Okay. And any other films at all?
Eva: *Titanic*.
I: Okay, so tell me a little bit more about *Titanic*. How do you remember it?
Eva: They [Jack and Rose, the leading romantic couple] kind of don′t let anyone get in the way between them, and like I wish that's like what I done [laughs]. So like I′ve learnt from my mistake that not to do that again.

This final statement refers to Eva's account of having broken up with her boyfriend because of fear of what her friends and his might think about their relationship. Here she uses the relationship in *Titanic* as a model for understanding what went wrong in her relationship and how she might act differently in the future.

## Gendered accounts: presumed media influence on romantic and sexual relationships

Boys typically saw girls as being more directly influenced by the media (Kai II, Harvie; B.Ya.4a). Norman (II) said: ‘I think boys see it [the media], and someone's like that, ‘you′re really stupid I won′t do that’ [they will not be influenced by what they see], but girls will see someone acting, like they′ll say, ‘I′ll act like that’’. Bruce and Calum (B.Ya.3a) said that because girls get their expectations from TV – in particular, from ‘love movies’ and ‘happily ever after’ movies – this is what they expect from a relationship. Frank (II) concurred, saying that the expectation that girls derive from TV is that relationships are going to be perfect. Mark (B.Ya.3b) declared: ‘They′d maybe change what they think from media … girls do get ideas from TV, but I don′t know what – you have to ask them’. A boy's group discussion said:

I: Do you think that girls get any of their expectations about relationships from the media?
Kai: Yeah. ‘Coz there's like chick flicks.
Martin: Paris Hilton. *Titanic*.
Kai: Aye, *Titanic*, actually.
Martin: Aw, I like that film.
Kai: Aye, shut up*. The Notebook, Love Actually* …
Adam: *Dirty Dancing*.
Martin: I like that film, too.
Kai: Aye, I bet you do.
I: And so what kind of relationship is portrayed in these films?
Kai: Lovey-dovey, run away, kiss each other …
Adam: Float about on bits of driftwood. Watch your boyfriend floating down to the bottom of the sea (B.Ya.3e)

Take Eric and Dave:

Eric: If they see a nice lassie on the TV and then see a nice boy they′re going to try and act the way they act to go out with them.
Dave: And you watch programmes where their pals give them advice and then the lassies who watch it will take that advice and try and use it. (B.Ya.3c)

Participants in B.Ya.4a also thought that girls would learn from programmes such as *Skins* and *Shameless*: that is, ‘how to dae things, how to have sex if she's never had sex’ (Bryn). The rest of the boys’ account of girls’ expectations revolved around perfect relationships which, for some boys were understood as being unrealistic expectations. Boys in B.Ya.3a said girls’ expectations were from ‘love movies’, ‘happily-ever-after’, *90210*, Disney and ‘mushy movies’. Frank said girls’ media-derived expectations were that ‘love conquers all’ and perfect relationships are about ‘true love, feeling protected and safe and happy’. Kai (II) thought that girls get ‘too influenced by things and have high expectations and you end up being disappointed’.

What of the girls’ accounts of presumed media influence? Of those participants who discussed the issue, most declared that boys derived their expectations about relationships from pornography. Girls from G.Ro.4a and G.Ro.4b were typical of this view. Some thought this to be more likely if the boy had not been in a relationship before; others said that boys expect girls to ‘always do what they [boys] want, that we should be easy’. This opinion, they suggested, derived from pornography. However, the girls assured the interviewer that they felt no pressure to act like a porn star for boys. Debbie (G.Ro.4b) said that ‘50 per cent’ of men watch porn daily. When asked how that made them feel she said: ‘Doesn′t really bother me – they always look so perfect, or the illusion of perfect. But I know boys who look for personality’. For Debbie, therefore, personal experience counteracts the presumed influence of media. Heidi (II) told of how boys in her school year download pornography to their mobile phones. Girls, on the other hand, did not watch porn, she said. She made a clear distinction between boys’ expectations and what boys feel they deserve: pornography may change what boys feel they deserve, and what they want but because it is so clearly fantasy and separated from real life, the boys would be ‘aware that it's not really possible’. Rhona (II) seemed more certain that the pornography that boys watch does influence their expectations of girls: ‘They kind of like more expect it from you, like you should be doing what these people are doing on these videos or photos or whatever they′re looking at’.

The analysis of the opposite-sex's expectations of sexual/romantic relationships and the perceived source of those expectations revealed the participants′ wish to belong and to be accepted. They appear to be attempting to understand what the rules and roles of relationships are, drawing heavily on what media content they presumed the opposite sex was exposed to. Many participants reported that the media played an important role in forming their expectations of what a romantic and sexual relationship is and how they should behave, but the participants thought it played a more important role in how the opposite sex perceived relationships. This was in sharp contrast to the media's perceived lack of influence in shaping drinking behaviour, to which we now turn.

## Gendered accounts: media portrayals of drinking and sex

Consider, first, how alcohol featured in the participants’ lives, regardless of whether or not they themselves drank. (Throughout their accounts there was a tacit assumption that to drink meant to get drunk.) As with media-use, alcohol loomed large, but the associations that they made between media-influence and alcohol were far fewer than the associations that (as reported above) they made between the media and relationships. In short, they very rarely engaged with this issue: in fact they often responded with a straightforward no to questions directed towards exploring their ideas of what was gender-appropriate drinking behaviour had been influenced by the media. Probing further was fruitless: they re-affirmed their original statement of ‘no, it isn′t the media’; ‘it's more peers’ or ‘it just happens’, as though it were an inevitable part of their life.

The diversity of experiences of alcohol consumption in the sample was striking. Whereas some described their average weekend simply as being ‘drunk’, others had never drunk alcohol and knew of no-one who had. Some seemed to be in no hurry to grow up. ‘Growing up’ was an allusion to having sex and drinking alcohol:

I: How much time do you spend with your parents?
Eva: Recently quite a lot. Cause my friends sort of do stuff that I can′t be bothered doing half the time. I go to the cinema with my mum most of the time.
I: So what kind of things are your friends doing that you don′t want to do?
Eva: Going out to the park drinking and stuff like that.
I: Oh, I see. And that's just something that you′re not really interested in?
Eva: No.
I: Is your friendship affected by that?
Eva: Sort of, it's split me away from two friends but my other friend, she doesn′t like to do it either. So like on a Saturday night I′ll go stay with her. But it's kind of caused a divide between a couple of us. (II)

Even if they were not yet drinking, it was presumed that it would only be a matter of time until they were of an age to do so. For many male participants, drinking alcohol was a sign of maturity that alluded to sexual activity. In discussing the connection between drinking alcohol and sexual behaviour, the girls typically referred to their own or other girls’ behaviour. Talking in more general terms, Sarah said:

I mean, like, people in our year that have probably already slept with somebody think it's not that big a deal, but like, see when you′re like, you′re not drunk, you can say like, ‘no’ to it [sex] and that, if you don′t want to do it, but see like when you are drunk, you don′t really have control of your body and you just say ‘yes’ to everything. (G.Ya.3b)

Bryn offered a bolder comment about his perceptions of the link between girls’ drinking and sexual behaviour: ‘They′re wee sluts when they drink’. Most responses from boys about boys concerning drinking and sexual behaviour were speculative, the exchange below being typical:

Darren: When you′re, like when you′re drunk you′re like more relaxed and let go and –
Calum: Don′t care what you′re doing.
Darren: – go with the flow I think. You just –
Calum: It's like other people go and have sex and then they′re all like that, ‘on you go it's like fun’. And then you just go like that, ‘all right’.
I: So you don′t really think about it as much then?
Calum: Aye, exactly, ‘coz, like when you′re drunk you kind of lose some brain cells and you can′t think as well. But when you′re like not drunk you actually know what you′re doing and where you′re going and what's going to happen. (B.Ya.3a)

In the excerpt below, Rhona (II) discussed the sexual behaviour of drunken boys whom she knew:

I: What do they [boys] get up to when they′re drunk?
Rhona: I′m not really sure that – well, boys kind of take advantage of the girls if they′re a bit drunk and stuff like that. Like, people you wouldn′t normally get together with, tend to, just because their judgments are clouded and stuff like that. But I don′t think the guys necessarily mean to take advantage of them, it's just they think ‘they′re up for it so, let's go for it.’

More generally, several participants told interesting stories of acting ‘out of character’ in a wider sense when drunk. In the excerpt below girls are discussing other girls’ behaviour:

Kate: I think some people just, like, pretend to be drunk so that they can get away with everything and –
Jill: They′re drunk a wee bit and then they just start acting it.
Kate: Yeah.
I: So why are they pretending to be drunk?
Jill: To show off.
Susan: To show off and look cool and that.
I: Acting in a way that they couldn′t act when they′re sober?
Susan: Mhmm.
I: And what would that be? What would they be doing?
Susan: Like shouting and walking about funny and –
Jill: Pure, like, hanging on to the boys and that and like –
Kate: Just being, like, too confident
Sarah: Like, thinking too high of yourself and that and … just like … you kinda like, yeah, you practically, like, shout everything.
Jill: Basically like you walk about yourself, like –
Susan: You pretend that you can say anything you want, that you can get away with it, like … people think you′re drunk, OK, it's no big deal or that, what you say. (G.Ya.3b)

It might be inferred that pretending to be drunk is a licence to act differently, to try out new behaviour, be someone else, say what cannot be said in normal daily exchanges and experiment with different identities. This seems to be an important element of drinking alcohol, allowing one some inconsistency in self-presentation and sexual behaviour and a chance to gauge how others react and reject ownership of that behaviour if reactions are negative.

How did the media influence the relationship between alcohol and sexual/romantic behaviour? Participants′ typical understandings of media portrayals of girls′ drinking involved parties, sex and fighting. To some extent, girls′ reported real-life experiences of drinking that did correspond with these portrayals, except that there were fewer parties but drinking invariably involved socialising; there was less penetrative sex but drinking was often associated with romantic/sexual relations with the opposite sex; and there was less fighting but a lot of aggression. The participants′ general understanding of the media-portrayals of boys being drunk involved promiscuous sex and physical aggression. Again, it can be suggested tentatively that the real-life experiences of some of the boys′ drinking did correspond to the media-portrayals.^3^ Accounts of drunken sexual encounters and physical aggression were reported to be common outcomes of boys′ drinking. All this said, it bears repeating that the associations that they made between media influence and alcohol were far fewer than the associations that they made between the media and relationships.

## Discussion and conclusion

This study has explored how media portrayals of teenagers′ drinking and romantic/sexual relationships might inform or influence their construction of gendered identities. Some methodological asides are apposite. First, no data collection of actual media use was undertaken. Many of the data are in the form of teenagers′ generalised understandings, and their principal utility is to reveal underlying stereotypes that predominate in the accounts. Secondly, we discouraged disclosure of specific details about alcohol consumption and sexual/romantic relationships. Thirdly, questions on sexual/romantic relationships were prefaced by a comment about there being no wrong or right way of doing relationships, and that a relationship can be between a girl and a boy, boy and a boy, or a girl and a girl. Even so, it is probably the case that the participants shared the interviewer's assumption that the relationships being discussed were heterosexual. In future studies, with careful thought, the issue of sexual orientation could be given more prominence. Finally, the two schools had similar socioeconomic profiles. No socioeconomic data on individuals were collected. The group discussion participants were selected by teachers and appeared to be a mix of working-class and middle-class in both schools. However, out of necessity, the follow-up individual interviews from Rosefield High were all with middle-class girls, with the exception of one middle-class boy. This over-representation of middle class participants is important because the individual interviews enabled the collection of detailed and in-depth biographical data. Future research should prioritise recruiting participants from different socioeconomic backgrounds.

The rich qualitative data lend support to Milkie's (1999) presumed media influence theory, a nuanced application of symbolic interactionism. It supposes that the media are influential by virtue of one's expectations about how the media may have influenced other significant people in one's social group. The influence of the mass media is mediated by social relationships in complex ways. For this process to operate it was essential that participants were able to infer what was going on in the minds of others and, in particular, in the minds of the opposite sex. In this respect, it is important to re-emphasise here that participants were reporting on their awareness of what media their peers – particularly their opposite-sex peers – are exposed to. For example, the boys often reported that they knew which television soaps girls watched; the girls talked of boys watching pornography. Teenagers gleaned this information from discussing and watching media with friends and sometimes from overhearing others talking about media. In order to presume media influence on others, they had to both think they knew what the others had watched and to be sufficiently familiar with its content. These two considerations are significant for participants′ expectations of the opposite-sex's expectations.

While these teenagers reported that media presentations of gender-appropriate romantic and sexual relationships were influential, media presentations of alcohol consumption were less so. This was because participants had less firsthand experience of sexual/romantic relationships and they were less public, so they had less information to draw on. The media portrayals compensated for this. Put differently, media portrayals of activities that are more public – in this case, drinking alcohol – may merely complement other and stronger influences such as peers, family and personal experiences.

Thus far, an analytical distinction has been drawn between the two health-related forms of behaviour; alcohol use and sexual relationships. The participants in this study were able to report either about themselves or about others, as they attempted to perform gender appropriately through their sexual/romantic relationships and in drinking alcohol. The findings here focused mainly upon gender and strongly indicate that the construction of gendered identities is an important element of alcohol use and sexual behaviour.

In sum, the 13 to 15-year olds in these two Scottish urban areas reported the media to be influential in how they presented their gender-appropriate romantic and sexual relationships, but to be less influential in how they used and understood the meanings of alcohol. The processes of media influence appear complex but they seem to operate primarily through presumptions about how one's peers were influenced, thus supporting Milkie's hypothesis. Although the participants reported that the media did not influence their expectations of gender-appropriate drinking, the findings nonetheless suggest that the media can influence this behaviour. Teenagers may drink alcohol to help them connect with the opposite sex and the ways in which they do this may be shaped by their expectations of what is gender-appropriate, as learned from the media portrayals of those behaviour. What emerged strongly was the salience for these teenagers of constructing a gender-appropriate identity. They believed that certain kinds of drinking and sexual behaviour made them appear more or less feminine or masculine. The interconnectedness of forms of health behaviour, as understood through the lens of gender-identity construction, opens up a new conceptual space which could explain the interconnectedness of other kinds of health behaviour, and behaviour among other age groups.

## Notes

*Skins* revolves around the lives of a group of 16–19-year-old friends who live in Bristol. They are frequently portrayed drinking or taking drugs, and sex and relationship issues feature throughout. *Hollyoaks* is set in a fictional Chester suburb and is centred on the local college. The characters are generally in their late teens or early twenties. *Shameless* follows the lives of a ‘dysfunctional’ family who live on a working-class estate near Manchester. See Hartley (2011) for the media images used.

*Beverly Hills 90210* is an American drama series following the lives of a group of teenagers living in the wealthy, film star-studded community of Beverly Hills, California.

Media portrayals here refer both to the still images presented from *Skins, Hollyoaks* and *Shameless* and to general discussions about the media portrayals of this behaviour.
